# Development of automated metabolite control using mid-infrared probe for bioprocesses and vaccine manufacturing

**DOI:** 10.1093/jimb/kuae019

**Published:** 2024-06-11

**Authors:** Jennifer Reid, Manjit Haer, Airong Chen, Calvin Adams, Yu Chen Lin, Jim Cronin, Zhou Yu, Marina Kirkitadze, Tao Yuan

**Affiliations:** Global Bioprocess Development, Sanofi, Toronto, ON M2R 3T4, Canada; Analytical Sciences, Sanofi, Toronto, ON M2R 3T4, Canada; Global Bioprocess Development, Sanofi, Toronto, ON M2R 3T4, Canada; Global Bioprocess Development, Sanofi, Toronto, ON M2R 3T4, Canada; Analytical Sciences, Sanofi, Toronto, ON M2R 3T4, Canada; Mettler Toledo, Columbia, MD 21046, USA; Global Bioprocess Development, Sanofi, Toronto, ON M2R 3T4, Canada; Analytical Sciences, Sanofi, Toronto, ON M2R 3T4, Canada; Global Bioprocess Development, Sanofi, Toronto, ON M2R 3T4, Canada

**Keywords:** Automation, Mid-infrared spectroscopy, Process analytical technology, Bioprocess development, Fermentation, Process information management system

## Abstract

Automation of metabolite control in fermenters is fundamental to develop vaccine manufacturing processes more quickly and robustly. We created an end-to-end process analytical technology and quality by design-focused process by replacing manual control of metabolites during the development of fed-batch bioprocesses with a system that is highly adaptable and automation-enabled. Mid-infrared spectroscopy with an attenuated total reflectance probe in-line, and simple linear regression using the Beer-Lambert Law, were developed to quantitate key metabolites (glucose and glutamate) from spectral data that measured complex media during fermentation. This data was digitally connected to a process information management system, to enable continuous control of feed pumps with proportional-integral-derivative controllers that maintained nutrient levels throughout fed-batch stirred-tank fermenter processes. Continuous metabolite data from mid-infrared spectra of cultures in stirred-tank reactors enabled feedback loops and control of the feed pumps in pharmaceutical development laboratories. This improved process control of nutrient levels by 20-fold and the drug substance yield by an order of magnitude. Furthermore, the method is adaptable to other systems and enables soft sensing, such as the consumption rate of metabolites. The ability to develop quantitative metabolite templates quickly and simply for changing bioprocesses was instrumental for project acceleration and heightened process control and automation.

**One-Sentence Summary:**

Intelligent digital control systems using continuous in-line metabolite data enabled end-to-end automation of fed-batch processes in stirred-tank reactors.

## Introduction

Accelerated vaccine bioprocess development requires the integration of intelligent digital technologies and networks to better understand, control, and predict fermentation processes in real-time. Leveraging process analytical technologies (PAT) connected to digital networks (i.e. process information management systems, PIMS) enables robust and adaptable automation capability that has become a major focus in the digital transformation of pharmaceutical manufacturing (Arden et al., 2021; Gerzon et al., 2022).

PAT has been an important mechanism to design, analyze, and control pharmaceutical manufacturing processes for many years through the measurement of critical process parameters and quality by design processes (FDA, 2004). Maximizing crude harvest yield from stirred-tank reactors is a key component in the overall productivity of manufacturing processes. However, it has also been described as the most challenging unit operation for process control (Alford, 2006). Most bioreactor and fermenter distributed control systems routinely manage common process parameters (e.g. pH, dissolved gas control, temperature); however, controlling nutrient levels is not a streamlined process. Nutrient control is further complicated due to the inherent high process variability of biological systems and the need to maintain a sterile environment. For example, there are relatively few real-time direct measures available from stirred-tank reactors, such as withdrawing samples to measure nutrients with off-line bioanalyzers, as these methods are resource-consuming, inconsistent, risk biohazard exposure to the operators, and also risk contamination of the bioreactor or fermenter.

Fortunately, nutrient levels and other variables that influence growth and yield, can be measured in real-time with spectroscopic methods in-line (André et al., 2015). Chinese hamster ovary (CHO) cell culture process improvements have demonstrated the use of Raman spectroscopy (Gibbons et al., 2023) as well as near infrared (NIR) spectroscopy (Kozma et al., 2019) to determine metabolite concentrations in stirred tank bioreactors in the biopharmaceutical industry. Both methods produce large spectral data sets in real-time that are not readily interpretable without multivariate modeling and may be complicated by scattering from suspended solids (e.g. cells, microcarriers) and bubbles from aeration (Esmond-White et al., 2022). While the success of Raman for metabolite control in CHO cell culture processes has been reported (Gibbons et al., 2023) and commercialized (Yamanaka et al., 2021), unfortunately, microbial-based fermentation presents a number of different challenges for in-line spectroscopy-based PAT. These challenges include high rates of aeration creating a large number of bubbles that can scatter the Raman signal (Esmond-White et al., 2022), dark media colors that can absorb the Raman signal, generally more intense fluorescence compared to CHO cell cultures (at 785 nm excitation), exponentially changing cell mass (more scattering centers), and shorter fermentation durations that place a premium on speed of measurements for process control (Farrell et al., 2012).

In reports of direct comparison for metabolite analysis of mammalian cell cultures, Raman, and mid-infrared (MIR) spectroscopy perform similarly overall, albeit each has their own product-specific benefits and limitations (Graf et al., 2021). MIR spectroscopy coupled with a diamond attenuated total reflectance (ATR) probe, however, allows the PAT to overcome the challenges related to measuring metabolites from microbial fermenters in-line (Doak & Phillips, 1999) and from samples off-line (Sivakesava et al., 2001). The short effective pathlength of the ATR measurement eliminates interference from the scattering of bubbles and suspended solids (Doak & Phillips, 1999), which is critical for monitoring microbial fermentation *in situ* where cell mass concentrations are rapidly changing and receive high aeration (Farrell et al., 2012). MIR with an ATR probe also maintains a constant, short pathlength, enabling the collection of high-quality infrared spectra in water and other strongly absorbing solvents (Doak & Phillips, 1999). Furthermore, MIR peaks are fundamental vibrations associated with the functional groups of a component, as opposed to NIR, in which the peaks are highly overlapped (overtones and combination bands) (Metcalfe et al., 2020). Generally, a Beer-Lambert Law approach can be used to correlate MIR peak intensities to the concentration of a given component (Doak & Phillips, 1999). Trending individual peak intensities over time to monitor common metabolite concentrations can be successful in microbial fermentations, primarily due to the simplicity of the media and that many microbial recombinant products are intercellular and therefore do not interfere with the quantification of extracellular metabolites. Tracking metabolite concentrations based on discrete infrared peaks eliminates the need for time-consuming multivariate models and fits well with accelerated pharmaceutical process development. For these reasons, MIR coupled with an ATR probe was used in this evaluation as an in-line PAT for fermentation.

In addition to the benefits of passive metabolite monitoring, the digital networking of the MIR in-line PAT into a PIMS enables continuous in-line data to automate feed-back control and maintain desired metabolite setpoints without operator intervention (Gibbons et al., 2023). The present study implemented an automated metabolite feed-back control system to improve an upstream fermentation process in stirred-tank fermentors, a process that is representative of common microbial fermentation in the pharmaceutical industry. The MIR in-line PAT was coupled to a PIMS for end-to-end automation that was rapidly adaptable to process development: different production scales, media compositions, growth profiles, and metabolite setpoints across and within fermentation runs. The two main nutrients developed for independent control were glucose and monosodium glutamate (MSG, also referred to as glutamate) as commonly used carbon and nitrogen sources, respectively. The end-to-end automation provided by the digital PAT and PIMS network significantly improved process control by 20-fold, which led to a 10-fold increase in the productivity of a small-molecule drug substance and provided real-time metabolite consumption rate data for greater process understanding.

## Materials and Methods

### Fermentation and PIMS

All materials used were produced in-house (Sanofi, Toronto, Canada). Recombinant organisms were grown in a complex medium that includes glucose, monosodium glutamate, and yeast extract. Cultures were operated in batch mode until glucose and glutamate concentrations decreased to 20 g/L, which triggered the initiation of two independent supplemental feeds comprised of 600 g/L glucose or 400 g/L monosodium glutamate, termed fed-batch control. In this study, 2-L fermenters (Biostat B-DCU, Sartorius) equipped with Rushton impellers and baffles were operated at a percentage working volume between 40% and 70%. Antifoam (4% Biospumex, PMC Ouvrie) was added throughout fermentation as required and not factored into the measurements of glucose and glutamate by the MIR in-line PAT. Air was supplied at a volumetric flow rate that ranged from one to two volumes of gas per volume of culture media per minute (1–2 vvm). The temperature was controlled by a water jacket at a constant setpoint. Reverse phase liquid chromatography (RPLC) quantitative analysis was used to estimate the yield of the small-molecule drug substance. All off-line samples were taken aseptically from 2-L fermenters during the fermentation. Metabolite concentrations were analyzed using the supernatant fraction of the sample, tested with a Cedex Bio Analyzer (Roche). Lucullus^®^ (Securecell, version 3.11.3) is a PIMS client/server application for the acquisition, display, archiving, and reporting of information from a wide variety of systems, including discrete pumps, fermenters, and PAT solutions. Continuous data was transferred from iC IR™ software (see *Mid-Infrared Sensor* section below) to the PIMS using OPC UA. PIMS was used to control feed pumps based on a proportional-integral-derivative (PID) controller and metabolite setpoint; only the proportional term was required.

### Mid-Infrared Sensor

MIR spectra were recorded using a ReactIR 702L (Mettler Toledo Inc., USA) equipped with a 9.5 mm-diameter DiComp ATR probe with a 1.5-meter fiber. The ATR probe was disinfected with 70% isopropyl alcohol prior to use. The probe was installed through the headplate of stirred 2-L fermenters that were sterilized by autoclaving. Fourier transform infrared spectroscopy spectra were collected in the range of 650 and 3000 cm^−1^ at 8 cm^−1^ resolution, with 512 spectral scans collected every 5 min using iC IR™ software (version 7.1). The absorbance of ultra-pure water was subtracted from the spectral data as ‘background’. The absorbance of glucose was measured nominally at 1035 cm^−1^ (location: 1058–978 cm^−1^, baseline: 1058–1004 cm^−1^), whereas the absorbance of glutamate was measured nominally at 1400 cm^−1^ (location: 1413–1393 cm^−1^, baseline: 1436–1379 cm^−1^). Glucose and glutamate absorptivities were calculated by comparing off-line Cedex Bio analyzer results with the associated peak intensities and averaging them over multiple observations. Beer-Lambert's Law was used for quantitation, where absorbance (A) and concentration (c) are linearly correlated:


\begin{equation*}
A = \ {\mathrm{\varepsilon lc}}
\end{equation*}

where: *A* = absorbance, *ε =* the molar attenuation coefficient or absorptivity of the attenuating species, *l =* length of the path light must travel in the solution; pathlength is related to the design of the diamond ATR probe and is constant, *c =* the concentration of the attenuating species.

Absorptivity and the empirically determined εl terms for glucose and glutamate were calculated using multiple grab samples from two independent fermentation runs. Linear regression, based on Beer-Lambert's Law, was used within iCIR software (version 7.1.91) to compute the concentration of glucose and glutamate in line and real-time with the following equation (multivariate analysis was not required): metabolite concentration (g/L) = εl × Absorbance + constant. A constant was required to account for background absorbance due to other media constituents.


\begin{equation*}
\textit{Glucose}\,\textit{concentration}\ \left( {g/L} \right)\ = 870.29\ \times \textit{Absorbance} + 6.955
\end{equation*}

\begin{equation*}
\textit{Glutamate}\ \textit{concentration}\ \left( {g/L} \right)\ = 526.196\ \times \textit{Absorbance} + 0.6888
\end{equation*}

### Reference Methods

Reference assays were used to measure pH, glucose, and protein concentration. A list of reference percentage (%) errors for various parameters, including temperature probe (5%) (Mettler Toledo), pH probe (5%) (Mettler Toledo), glucose (Cedex Bio Analyzer, Roche Applied Science) (10%), and glutamate (Cedex Bio Analyzer, Roche Applied Science) (10%), were represented as standard errors for each instrument previously used in current measurement practices. Each instrument provided measurements for each respective parameter to use as data to support the MIR calibration models. The percentage error of each method is incorporated to show the original degree of error provided by each of the instruments to consider the error of measurement in the respective MIR models.

### Statistics

Statistical analyses were performed in Prism (GraphPad, version 9.5.0). No data points were excluded, and sample size is described for each figure; ‘*N*’ refers to the number of independent experiments, and ‘*n*’ refers to the total number of biological measurements (not technical replicates). P values are displayed on all relevant plots. The feed pump rates were manually calculated in Excel (Microsoft 365) using either the off-line Cedex Bio analyzer or in-line PAT determined concentrations of glucose and glutamate prior to the implementation of end-to-end automation using PIMS (*described above*). Integration was used for quantifying the variation from the set point for Fig. 4E–G. Variation from the set point was calculated as the integral of the present value, using the set point as a baseline in Excel. If the present value was below the setpoint, the integral was calculated as an absolute value. The cumulative deviation represents the total shaded area shown in Fig. 4E is an increasing absolute sum as a function of time (Fig. 4F) and as a total absolute sum from 48 hrs of fermentation data (Fig. 4G). All experiments in this dataset contain 48 hrs of data, starting from the beginning of a fermentation run. For Fig. 4I, the consumption rate was calculated with the following equation:


\begin{equation*}
{{C}_t} = \frac{{{{G}_{t - 1}} \times {{V}_{t - 1}} - {{G}_t} \times {{V}_t} + F \times S}}{I}
\end{equation*}

Where: *C_t_ =* consumption rate as a function of time (g/h), *G_t–1_ =* metabolite concentration of the second most recent measurement quantified by the MIR PAT (g/L), *V_t–1_ =* fermenter volume at the time of the second most recent measurement (L), *G_t_ =* metabolite concentration of the most recent measurement quantified by the MIR PAT (g/L), *V_t_ =* fermenter volume at the time of the most recent metabolite measurement (L), *F =* feed volume pumped into reactor between time ‘*t*–1’ and ‘*t*’ (mL), *S =* constant; concentration of metabolite in feed (g/mL), *I =* the interval of time between ‘*t*–1’ and ‘*t*’ in hours (h).

## Results

### Manual Control of Nutrients Led to Poor Process Control During Fermentation

**Fig. 1.**
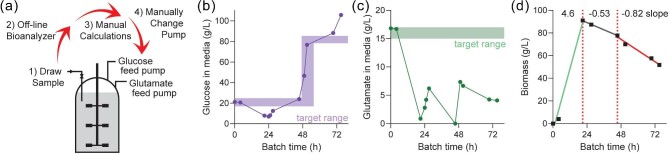
(**a**) Schematic of steps involved in manual control of metabolite levels during fermentation. Representative process control of glucose (**b**) and glutamate (**c**) during manual control of nutrients during fermentation, using off-line samples and bioanalyzer. Rectangles indicates desired target setpoint ranges. (**d**) Biomass (wet cell pellet) during the same representative fermenter run. The slopes of the data were calculated from data of each 24 h period, indicated with dotted red lines.

An upstream bioprocess optimization project was initiated within Sanofi Vaccines for a recombinant organism that was cultured in a complex medium containing glucose, MSG, yeast extract, and trace elements in stirred-tank fermenters equipped with Rushton impellers and baffles. It was determined that glucose and glutamate would be independently controlled by independent feed pumps that would be continuously pumping at changing rates throughout fermentation to maintain different setpoints. Culture samples were drawn from the fermenter on a regular basis during regular business hours (8 hrs daily) and measured for glucose and glutamate concentrations with an off-line bioanalyzer (Fig. 1a). This data was inputted by process operators into an Excel table that automatically calculated the required feed pump speeds necessary to return the fermenter metabolite levels to the desired set point before the next sample would be taken. The feed pumps in the development labs then had to be manually changed. Unfortunately, this initial system was prone to error due to the length of delay between sampling and changing the pump speed, as well as the inability to adequately control metabolite levels overnight. Coupled to the non-linear and rapidly changing growth characteristics of the recombinant organism, this led to frequent over- and under-shooting of the desired metabolite levels (Fig. 1b and c). Poor metabolite control resulted in confounded process optimization studies. For example, a fermentation experiment with a glucose concentration setpoint increase (Fig. 1b) occurred simultaneously with accidental glutamate depletion (Fig. 1c). Therefore, the root cause of biomass loss toward the end of this experiment was difficult to determine (Fig. 1d). An automated and robust system that could handle rapidly evolving process parameters was therefore considered and implemented to improve process control and small-molecule drug substance development.

### Development of MIR PAT to Quantify Metabolites In-Line During Fermentation

**Fig. 2.**
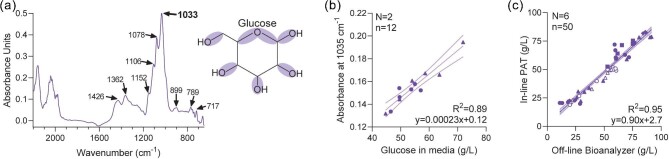
(**a**) MIR spectra of glucose dissolved in water, with the absorbance of water subtracted. Glucose chemical structure is shown with primary alcohol and ether bonds shaded. (**b**) Samples were drawn from two fermenter runs and the supernatant was analyzed off-line with a bioanalyzer and MIR with ATR probe. Linear regression statistics on plot, with 95% confidence interval shown. (**c**) Samples were drawn from six fermenter runs and the supernatant was analyzed off-line with a bioanalyzer, this was compared to the in-line MIR-based glucose concentration. Linear regression statistics on plot. Abbreviations: ATR, attenuated total reflectance; MIR, mid-infrared.

A MIR spectroscopy PAT with an ATR probe was evaluated for in-line process monitoring during fermentation due to the inherent simplicity of the modeling required; the concentration of attenuating species (i.e. chemical bonds) is linearly correlated to absorbance using Beer-Lambert's Law (*refer to Materials and Methods*). As such, the MIR spectra of glucose dissolved in water were measured to locate a narrow and intense region of absorbance specific to carbon-oxygen ether and alcohol bonds (Fig. 2a). It was determined that the absorbance at 1035 cm^−1^ was the strongest absorbing region and linearly correlated to the concentration of glucose in off-line samples. For this initial analysis, off-line samples were taken intermittently for up to 120 hrs of continuous fermentation, and the supernatant was measured using an off-line bioanalyzer and then freeze-thawed and centrifuged prior to MIR analysis (Fig. 2b; *R*^2^ = 0.89). The ATR probe from the MIR PAT was then installed into the fermenter and collected continuous spectral data throughout a fermenter run at a very early stage of the vaccine development project, as such the in-line glucose concentration was displayed on a laptop adjacent to the fermenter. Off-line samples continued to be taken for manual metabolite control. This single run was sufficient to create a quantitative template for glucose concentration that was used for a total of six independent fermentation runs where off-line samples were also taken (Fig. 2c). Linear regression of the in-line MIR PAT-based and off-line bioanalyzer-based glucose concentrations showed a significant positive correlation (*R*^2^ = 0.95). We considered the PAT sufficiently accurate so that off-line samples were no longer required to be drawn from the fermenter.

**Fig. 3.**
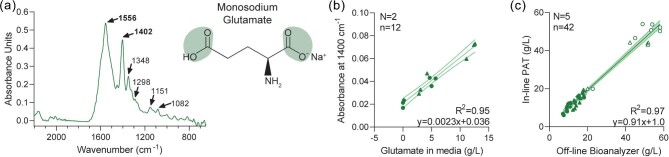
(**a**) MIR spectra of MSG (i.e. glutamate) dissolved in water, with the absorbance of water subtracted. Glutamate chemical structure is shown with carbonyl bonds shaded. (**b**) Samples were drawn from two fermenter runs and the supernatant was analyzed off-line with a bioanalyzer and MIR with ATR probe. Linear regression statistics on plot, with 95% confidence interval shown. (**c**) Samples were drawn from five fermenter runs and the supernatant was analyzed off-line with a bioanalyzer, this was compared to the in-line MIR-based glutamate concentration. The sixth fermenter run used in Fig. 2 did not contain glutamate. Linear regression statistics on plot. Abbreviations: ATR, attenuated total reflectance; MIR, mid-infrared; MSG, monosodium glutamate.

Similarly, glutamate concentration quantitation was evaluated by first measuring the MIR spectra of MSG dissolved in water to locate a specific and intense region of absorbance for glutamate attributable to carbonyl bonds (Fig. 3a). Both 1556 and 1400 cm^−1^ were strongly absorbing regions. The absorbance at 1400 cm^−1^ wavenumbers was selected for glutamate quantitation since it produced a sharper peak and was less likely to overlap with other media constituents. Importantly, the absorbance at 1400 cm^−1^ was highly correlated to glutamate concentration from off-line samples in parallel with glucose testing (Fig. 3b; *R*^2^ = 0.95). One single fermentation run was sufficient to create a quantitative template for glutamate concentration, in parallel with glucose, that was used for a total of five independent fermentation runs where off-line samples were also taken (the sixth run for glucose did not contain any glutamate). Similar to glucose, linear regression of the in-line MIR-based and off-line bioanalyzer-based glutamate concentrations exhibited a significant positive correlation that was considered sufficiently reliable so that off-line samples were no longer required (Fig. 2c, *R*^2^ = 0.97).

During these fermentation runs, the metabolite data was continuously generated but had not yet been digitally networked to a PIMS. However, a remote desktop connection to the laptop displaying the metabolite data was created, along with remote access to a PIMS that could control feed pumps. This meant that an operator was still required to remotely view the metabolite data and then input a pump speed process variable into the PIMS. This extended the control time beyond regular business hours but was difficult to routinely implement.

### Transition to Automated Control of Metabolites Improves Process Control During Fermentation

**Fig. 4.**
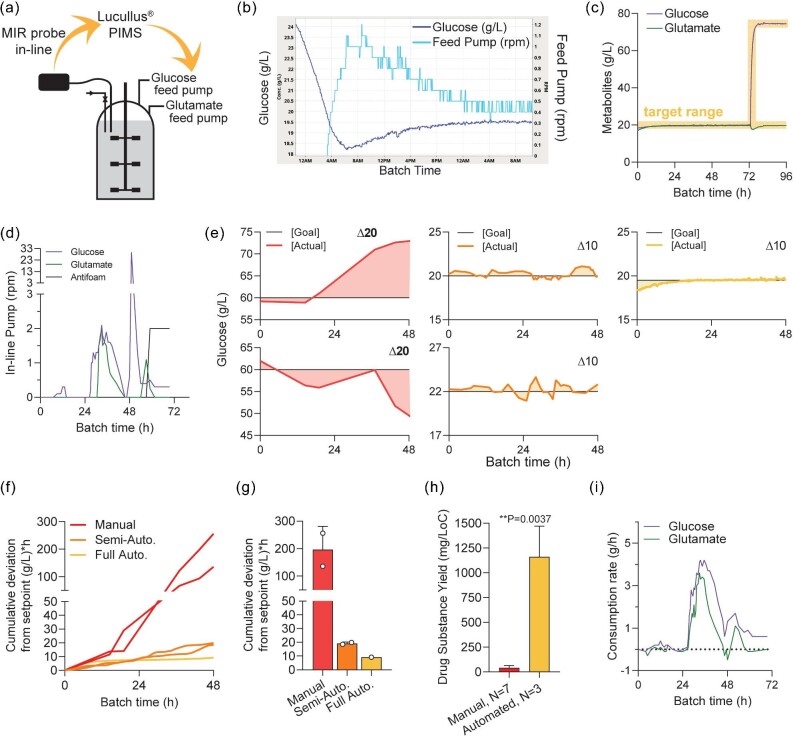
(**a**) Schematic of steps involved in automated control of metabolite levels during fermentation. (**b**) In-process automation raw values of glucose feed pump (rpm) during overnight working hours. (**c**) Representative process control of glucose and glutamate during fully automated control of nutrients during fermentation. Shading indicates desired target setpoint ranges. (**d**) In-process raw values of feed pump settings during the same fermentation run shown in Fig. 4c. (**e**) In-process glucose testing of five independent runs where manual control (*N* = 2, red), semi-automated control (*N* = 2, orange), and fully automated control (*N* = 1, yellow) are shown. Black line indicates setpoint and shaded areas indicates the integral area used for cumulative deviation from setpoint calculations. Note that ‘Δ20’ and ‘Δ10’ indicates y-axis ranges of 20 g/L and 10 g/L, respectively. (**f**) Cumulative deviation from setpoint using the sum of the integral of glucose concentration (shown in Fig. 4E) with the setpoint as a baseline. Refer to *Materials and Methods* for further calculation descriptions. (**g**) The total sum of glucose cumulative deviation from setpoint using the final sum of the integral of glucose concentration (shown in Fig. 4E) with the setpoint as a baseline. Refer to *Materials and Methods* for further calculation descriptions. (**h**) Representative drug substance yields from processes that used manual or automated metabolite control. Unpaired two-tailed *t*-test. Mg/LoC—milligram per Liter of Culture. (**i**) Consumption rate soft sensor using in-line metabolite data and PIMS-based calculations. Refer to *Materials and Methods* for further calculation descriptions. Abbreviation: PIMS, process information management system.

To complete the end-to-end automation of the feedback process, the laptop collecting MIR spectra and calculating metabolite concentrations was digitally networked to the PIMS, known as Lucullus^®^ (Fig. 4a). Lucullus^®^ used PID controllers to control the feed pump speeds automatically and continuously, which led to six main benefits. First, no operator intervention was required to control metabolite levels throughout the fermentation runs, nor were samples required to be drawn, which reduced the risk of exposure of operators to fermenter contents as well as fermenter contamination. Second, this automation enabled overnight starts of fed-batch processes, such that when the glucose concentration was rapidly decreasing, the PIMS was able to initiate glucose feeding by increasing the pump speed at approximately 3:00 AM (Fig. 4b). Third, metabolite level control enabled the structured design of experiments runs to be completed successfully, since confounding factors were reduced (Fig. 4c, as compared to Fig. 1a and b). Impressively, automation was able to hold the metabolite levels steady (Fig. 4c) despite fluctuating metabolite consumption rates during the same run (Fig. 4d). Fourth, in order to quantify the improvement of process control, the ‘accumulation of error’ was calculated using the glucose concentration interval (*refer to details in Material and Methods*). This enabled direct comparison of the impact of automation by comparing manual control, semi-automated, and fully end-to-end automated metabolite control (Fig. 4e; integral shown as shaded areas). The cumulative error of glucose concentration deviating from setpoint shows the dramatic 20-fold reduction of error when using automated systems along the process (Fig. 4f) and as a whole (Fig. 4g). The fifth benefit of end-to-end automation was the completion of the design of experiment studies that enabled a significant, order of magnitude, increase in productivity of the small-molecule drug substance (Fig. 4h). Finally, the sixth benefit was the additional data and insights gained from having in-line metabolite data, such as in-process consumption rate (Fig. 4i). Together, these results show that an intelligent digital network using MIR spectroscopy with an ATR probe for in-line metabolite control is highly useful and can be quickly implemented in pharmaceutical process development for fermentation.

## Discussion

The ability to develop quantitative templates for in-line metabolite analysis quickly and simply was instrumental for project acceleration, heightened process control, and automation of pharmaceutical bioprocess development for a small-molecule drug substance. We report the use of MIR spectroscopy, coupled to an ATR probe, to rapidly develop a quantitation method that calculated the concentration of two key metabolites without multi-variate analysis. With intuitive software and easy-to-use data in the hands of the user, the design of experiments could be completed and improved productivity and reduced resource requirements and risk.

Raman and NIR have generated a long and successful track record of in-line metabolite analysis, especially for mammalian cell culture, single-use bio-manufacturing, and routine production (Esmond-White et al., 2022). However, as previously described, the requirements for microbial process development (Farrell et al., 2012) vary substantially from CHO cell culture applications (Gibbons et al., 2023). In addition to the high background fluorescence associated with microbial fermentations and variable scattering from high aeration and rapidly changing cell mass, covering the entire fermenter with opaque material to eliminate ambient light that may interfere with Raman measurements can be impractical.

This study also presents preliminary data for the development of MIR spectroscopy as a potential soft sensor for fermentation during process development. Future directions include automation of triggering events once consumption rates have changed, as well as the potential to include MIR spectra in hybrid models for drug substance yield studies. These applications would not be possible without the digital network linking the PAT, fermenter, and other analyzers to a PIMS. Similar to previous editorials (Alford, 2006), the authors emphasize the key elements of digital transformation in pharmaceutical practices in order to accelerate vaccine bioprocess development.

## Conclusion

Continuous MIR spectral data (measured with an ATR probe) available to a PIMS enables real-time in-line monitoring and control of metabolites during bioprocess development. This study facilitated the transition from off-line to in-line monitoring and control with the networking of devices to a PIMS that could enable end-to-end automation of metabolite control. This reduced metabolite deviation by 20-fold and led to a significant 10-fold improvement of yield for a small-molecule drug substance. Future directions include applications with other organisms and media compositions, soft sensing to enable the determination of the appropriate time to collect crude harvest, as well as the use of MIR spectroscopy for hybrid models of fermenter processes.
